# A ^13^C isotope labeling method for the measurement of lignin metabolic flux in Arabidopsis stems

**DOI:** 10.1186/s13007-018-0318-3

**Published:** 2018-06-23

**Authors:** Peng Wang, Longyun Guo, Rohit Jaini, Antje Klempien, Rachel M. McCoy, John A. Morgan, Natalia Dudareva, Clint Chapple

**Affiliations:** 1 Department of Biochemistry, Purdue University, West Lafayette, IN 47907 USA; 2 Davidson School of Chemical Engineering, Purdue University, West Lafayette, IN 47907 USA; 3 Department of Horticulture and Landscape, Purdue University, West Lafayette, IN 47907 USA; 4 Purdue Center for Plant Biology, Purdue University, West Lafayette, IN 47907 USA

**Keywords:** Stable isotope labeling, Stem feeding, Lignin, Phenylpropanoids

## Abstract

**Background:**

Metabolic fluxes represent the functional phenotypes of biochemical pathways and are essential to reveal the distribution of precursors among metabolic networks. Although analysis of metabolic fluxes, facilitated by stable isotope labeling and mass spectrometry detection, has been applied in the studies of plant metabolism, we lack experimental measurements for carbon flux towards lignin, one of the most abundant polymers in nature.

**Results:**

We developed a feeding strategy of excised Arabidopsis stems with ^13^C labeled phenylalanine (Phe) for the analysis of lignin biosynthetic flux. We optimized the feeding methods and found the stems continued to grow and lignify. Consistent with lignification profiles along the stems, higher levels of phenylpropanoids and activities of lignin biosynthetic enzymes were detected in the base of the stem. In the feeding experiments, ^13^C labeled Phe was quickly accumulated and used for the synthesis of phenylpropanoid intermediates and lignin. The intermediates displayed two different patterns of labeling kinetics during the feeding period. Analysis of lignin showed rapid incorporation of label into all three subunits in the polymers.

**Conclusions:**

Our feeding results demonstrate the effectiveness of the stem feeding system and suggest a potential application for the investigations of other aspects in plant metabolism. The supply of exogenous Phe leading to a higher lignin deposition rate indicates the availability of Phe is a determining factor for lignification rates.

**Electronic supplementary material:**

The online version of this article (10.1186/s13007-018-0318-3) contains supplementary material, which is available to authorized users.

## Background

Metabolic fluxes quantify the activities of biochemical networks that functionally integrate metabolites, enzymes, and their interactions [1]. Mathematical analysis of metabolic fluxes is becoming increasingly attractive to assess the responses of metabolic pathways in plants to genetic or environmental perturbations [2]. Stable isotope labeling experiments coupled with mass spectrometry (MS) quantification have been used to measure the flux within metabolic networks in various plant systems and expand our knowledge of metabolism and the control of flux [3, 4]. For example, Szecowka et al. [5] supplied ^13^CO_2_ to intact Arabidopsis rosettes to estimate intracellular fluxes of photosynthesis and central carbon metabolism. Developing seeds of Arabidopsis, oilseed rape, and *Brassica napus* were fed with ^13^C labeled glucose or sucrose to elucidate fluxes in lipid synthesis and other primary metabolism [6–8]. Flux analysis has also been applied to unravel phenylpropanoid metabolism in plants. Matsuda et al. utilized deuterium labeled Phe to analyze metabolic flux towards defense-related products in potato tubers upon wounding stress or elicitor treatment [9–11]. Similarly, labeled Phe was provided to petunia flowers to address flux distribution through CoA-dependent and -independent pathways for benzenoid biosynthesis [3, 12]. A recent study with labeled glucose revealed that expression of the Arabidopsis transcription factor MYB12 induces primary and specialized metabolism to enhance flavonoid production in tomato fruits [13]. In contrast, there have been no measurements of metabolic flux into lignin, the most abundant carbon sink derived from phenylpropanoid metabolism.

Lignin is a heterogeneous aromatic polymer that constitutes approximately 20–30% of carbon fixed by land plants [14]. Deposited together with polysaccharides in the plant secondary cell wall, lignin provides mechanical strength and hydrophobicity for plants to stand upright and to transport water and nutrients through vascular structures [15]. Although important for plants, lignin is a major recalcitrance factor for forage digestibility, efficient paper-pulping, and biofuel production [16]. Alteration of lignin content and monomer composition in natural mutants and genetically engineered plants improves the efficiency of utilization of lignocellulosic biomass [17, 18], and has thereby motivated many studies of lignin biosynthesis and its regulation. Lignin in dicots is derived mainly from three monomers, *p*-coumaryl alcohol (H lignin), coniferyl alcohol (G lignin), and sinapyl alcohol (S lignin), all of which are synthesized from the phenylpropanoid pathway (Additional file 1: Figure S1) [15, 19, 20]. Phenylpropanoid metabolism starts with deamination of phenylalanine (Phe) by Phe ammonia lyase (PAL), the ring of which is subsequently hydroxylated by three cytochrome P450 enzymes [21] and *O*-methylated [22, 23], with some of these steps occurring at the level of CoA and shikimate esters of the hydroxycinnamic acids [24–26]. Ultimately, CoA thioesters are the substrates for reduction to their corresponding aldehydes and finally the alcohols commonly known as monolignols. Despite a comprehensive knowledge of the kinetics of most of the lignin biosynthetic enzymes and steady-state accumulation of metabolites involved, we lack systematic and quantitative measurements of the carbon flux into and within this branched metabolic pathway. Accurate quantification and prediction of lignin biosynthetic flux *in planta* will guide our understanding of controlling steps in the pathway and facilitate future genetic manipulations of lignification.

Reliable estimation of phenylpropanoid flux towards lignin and further analysis of flux regulation depend on precise quantification of phenylpropanoids and their isotopologues from appropriate plant tissue fed with isotope-labeled precursor. Arabidopsis stems deposit lignin at substantial levels and thus make a good system in which to analyze lignin biosynthetic flux not only in wild-type plants, but also in genetically modified plants with perturbations in Phe synthesis or lignin biosynthesis itself [19, 27]. Plants transport water and nutrients via the transpiration stream, which continues to function after plant organs have been removed from the parent plant. This property permitted experiments in which isotope-labeled amino acids or NH_4_NO_3_ were fed to white lupin, wheat, poplar, and Arabidopsis to study nitrogen transport and metabolism [28–31]. Similarly, stable isotope feeding assays of excised Arabidopsis leaves or whole rosettes were developed for the investigation of carbon partitioning in sink and source leaves [32]. In this study, we fed excised inflorescence stems of Arabidopsis with ^13^C_6_-ring labeled Phe ([^13^C_6_]-Phe) to trace phenylpropanoid metabolism. Our recently developed liquid chromatography coupled with tandem MS (LC/MS–MS) based phenylpropanoid metabolic profiling method provides fast and comprehensive analysis of pathway intermediates and end products [33]. All six isotopically-labeled carbons (^13^C) in the Phe ring are maintained in all intermediates and in lignin, allowing downstream labeled compounds to be distinctly detected and accurately quantified by MS. Furthermore, incorporation of label into lignin can be measured to estimate flux to the major pathway end product. We have optimized the feeding conditions and analyzed metabolite pool sizes and isotopic abundances, selected enzyme activities and lignin deposition during the feeding process. Over a time course of only 360 min, [^13^C_6_]-Phe rapidly labeled soluble intermediates and lignin, demonstrating the efficiency of the feeding strategy. These data together can be used for mathematical modeling of lignin deposition and the exploration of the regulation of lignin biosynthesis in plants [3, 9–12].

## Results

### Establishment of a stem feeding system using wild-type Arabidopsis

To perform stable isotope labeling experiments, we first established an experimental system using Arabidopsis stems that can be fed with exogenous [^13^C_6_]-Phe to synthesize labeled phenylpropanoids. We cut Arabidopsis stems under water and then transferred them into liquid Murashige and Skoog medium (MS medium) supplemented with [^13^C_6_]-Phe in 1.5 mL tubes (Additional file 1: Figure S2, see “Methods” section for details). Excising stems under water prevented cavitation of the xylem so that the transpiration stream would not be blocked.

**Fig. 1:**
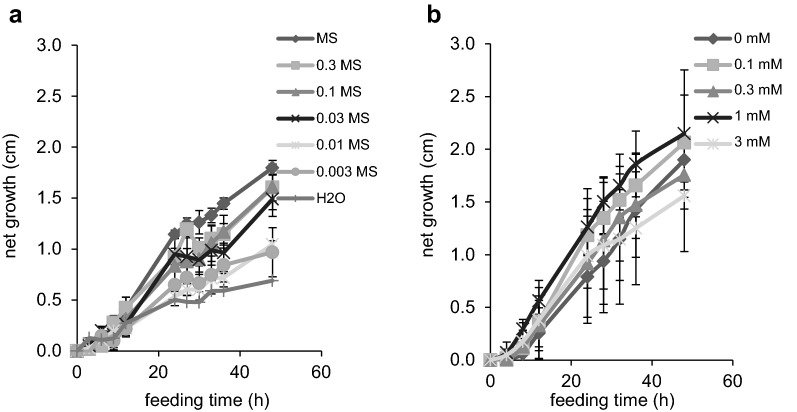
Growth of excised Arabidopsis stems in liquid medium. **a** Net growth in height of 4-week-old Arabidopsis stems excised and incubated in liquid MS medium of different strength for 48 h. Data represent mean ± standard deviation (n = 3). **b** Net growth in height of 4-week-old Arabidopsis stems excised and incubated in liquid MS medium containing Phe of different concentrations from 0 to 3 mM. Data represent mean ± standard deviation (n = 3)

To determine whether the excised stems are metabolically active we first asked whether the stems continue to grow in MS medium. When we assessed the growth of 4-week-old inflorescence stems, they elongated 2 cm in 48 h (Fig. 1a), the same as the reported growth rate of Arabidopsis stems in soil [34]. To test if the liquid medium is optimal for growth, stems were also grown in a series of diluted MS media. In the first 48 h after excision, stems grew in all conditions and showed the largest elongation in full-strength MS medium, suggesting that the medium is adequate and not toxic to the excised stems (Fig. 1a). In order to accurately estimate metabolic fluxes in stems, it is important that the supplied Phe does not perturb their growth [1], so we next analyzed the elongation of stems incubated in MS medium containing Phe of different concentrations. Supplying 0.1–3 mM of Phe had no effect on stem growth over 48 h (Fig. 1b). The amount of medium taken up by the stems was determined by weighing the medium remaining in the tube at various time points during the feeding process (Additional file 1: Figure S3). On average, each excised stem took up 1.41 ± 0.49 mg min^−1^, equivalent to 1.4 µL min^−1^ given that the density of medium is 1.0 g mL^−1^. Loss of medium through evaporation was negligible. These data indicate that we can feed Arabidopsis stems with exogenous Phe in conventional MS medium.

**Fig. 2:**
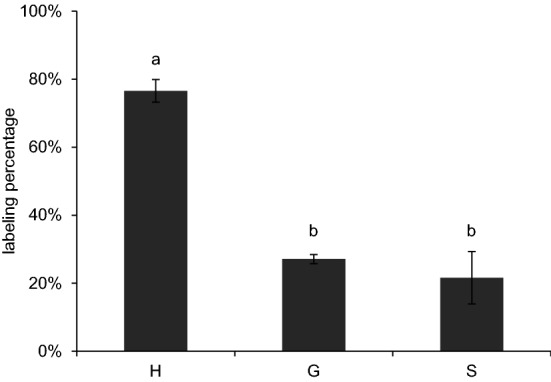
Labeling incorporation into lignin in stems fed with [^13^C_6_]-Phe. Labeling percentage of lignin monomers in stems fed with 0.3 mM [^13^C_6_]-Phe for 48 h were analyzed by DFRC/GC/MS. Data represent mean ± standard deviation (n = 3). The means were compared by one-way ANOVA and statistical difference by Tukey HSD test (*p* < 0.01) was indicated by letters

We next addressed whether the excised Arabidopsis stems can utilize the supplied [^13^C_6_]-Phe to synthesize labeled lignin. We fed stems with 0.3 mM [^13^C_6_]-Phe for 48 h and analyzed lignin monomers using the derivatization followed by reductive cleavage (DFRC) method coupled with gas chromatography (GC)/MS [35]. The DFRC method cleaves β-O-4 linkages within lignin and can be used to detect monolignols incorporated into the polymer derived from [^13^C_6_]-Phe. Monomers released corresponding to H, G, and S subunits from the stems fed with [^13^C_6_]-Phe were labeled to 77, 27, and 22%, respectively (Fig. 2). The higher labeling of H monomers released by DFRC relative to G and S may be due in part to the stems having larger pre-existing deposits of G and S lignin [19, 36], resulting in dilution of labeled monomers. The detection of ^13^C_6_-ring labeled lignin monomers in the stems indicates that the exogenous Phe is used for lignification and that ^13^C_6_-ring labeled lignin precursors are detectable by LC/MS–MS.

### Phenylpropanoid metabolism is not homogeneous along the stem

**Fig. 3:**
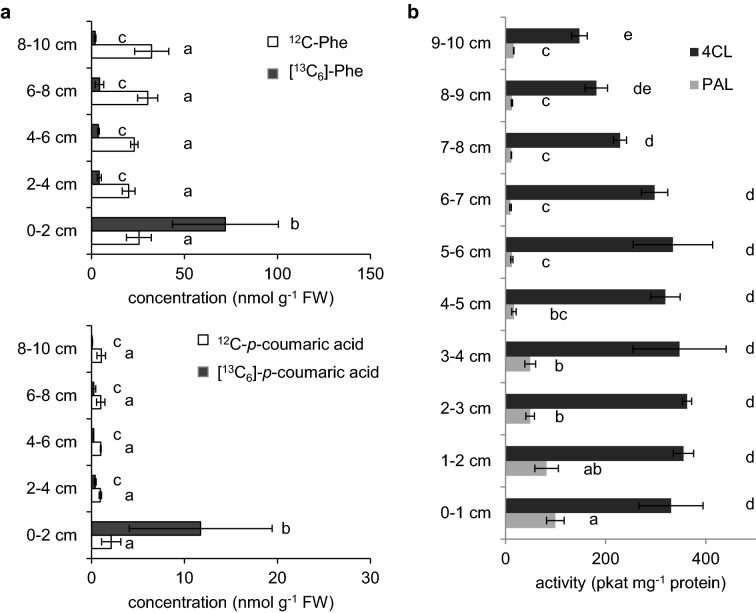
Phenylpropanoid metabolism along wild-type Arabidopsis stems. **a** Content of endogenous (white bars) and labeled (black bars) Phe and *p*-coumarate along the stems of 4-week-old wild-type Arabidopsis fed with 0.3 mM [^13^C_6_]-Phe for 6 h. Data represent mean ± standard deviation (n = 3). One-way ANOVA was used for unlabeled or labeled metabolites and statistical difference by Tukey HSD test (*p* < 0.01) was indicated by letters. **b** Activities of PAL and 4CL from base to top along the stems of 4-week-old Arabidopsis without feeding. Data represent mean ± standard deviation (n = 3). One-way ANOVA was tested for PAL and 4CL assays respectively and statistical difference by Tukey HSD test (*p* < 0.01) was indicated by different letters

The Arabidopsis inflorescence stem is a heterogeneous organ, in which the metabolite levels and enzyme activities have been reported to vary along the stem developmental gradient [27, 36]. For reliable flux measurements, an experimental tissue with consistent cellular phenylpropanoid concentrations and enzyme activities is needed. To determine the extent of heterogeneity of the Arabidopsis primary stem and find the best tissue for future experiments, we analyzed the concentrations of phenylpropanoids and their labeling percentage along the stems after feeding 0.3 mM of [^13^C_6_]-Phe for 360 min. The levels of endogenous compounds are similar along the stems (Fig. 3a), whereas, the concentrations of labeled compounds were much higher near the base of the stem than in the top. Over the 6-h period [^13^C_6_]-Phe was accumulated to 72 nmol g fresh weight (FW)^−1^ in the basal 2 cm, but only reached 4 nmol g FW^−1^ above 2 cm. Similarly, the concentration of [^13^C_6_]-*p*-coumarate was 12 nmol g FW^−1^ at the base, but only 0.4 nmol g FW^−1^ nearer the top. The high concentration of downstream intermediates including *p*-coumarate suggests that in the basal stem, exogenous Phe is actively sequestered from the transpiration stream, and possibly that enzymes in this region are more active in the consumption of Phe for lignin production than in upper parts. These data indicate that the basal 2 cm stem fragment appears to be an appropriate tissue in which to measure phenylpropanoid metabolic flux.

In addition to metabolite analysis, PAL and 4-coumarate: CoA ligase (4CL) enzyme activities were measured to assess the heterogeneity of lignin biosynthetic enzymes along the Arabidopsis inflorescence stem axis (Fig. 3b). PAL and 4CL both showed higher activities in the basal stems compared to those in the apical region. The enzyme activities together with the soluble metabolite pool sizes suggest that the stems have a larger phenylpropanoid flux in the base than the top, again consistent with the stem base being the best tissue for analysis. We thus chose to harvest this basal 2 cm fragment of stems for analysis in our feeding experiments.

**Fig. 4:**
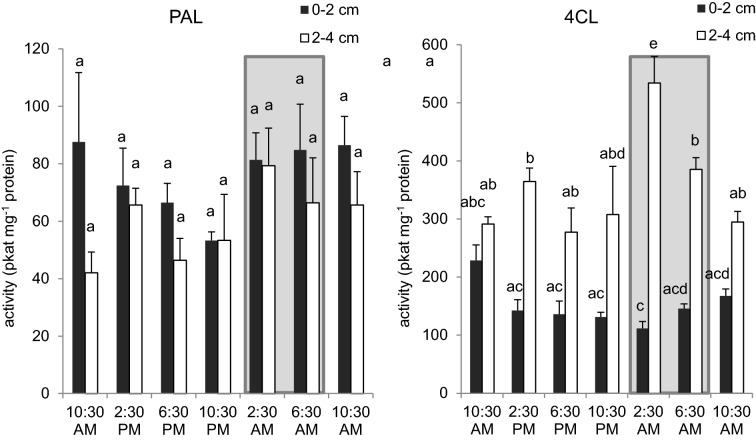
PAL and 4CL activities in Arabidopsis stems over a day–night cycle. Activities of PAL and 4CL from the basal 0–2 cm (black bars) or 2–4 cm (white bars) of 4-week-old Arabidopsis stems. Shaded area means stems were sampled at night. Data represent mean ± SD (n = 3). One-way ANOVA was tested for PAL and 4CL assays respectively and statistical difference by Tukey HSD test (*p* < 0.01) was indicated by different letters. Bars with a shared letter have no statistical difference from each other

As shown above, phenylpropanoid metabolism varies across the developmental profile of the Arabidopsis inflorescence stem, but it is also known that metabolite accumulation, enzyme activities and gene expression can exhibit diurnal fluctuations. Recent studies have shown that transcript abundance of lignin biosynthetic genes is higher during the night in Arabidopsis [37] so we determined PAL and 4CL enzyme activities over a 24-h period. We found that both enzymes were not statistically different in the basal stem 0–2 cm fragments (Fig. 4). However, 4CL activity was higher at night compared to that in the day in the 2–4 cm fragment of the stem. These results indicate that it is appropriate to conduct our feeding experiment during the day and on 0–2 cm fragments.

### Soluble phenylpropanoids are rapidly labeled in stems fed with [^13^C_6_]-Phe

**Fig. 5:**
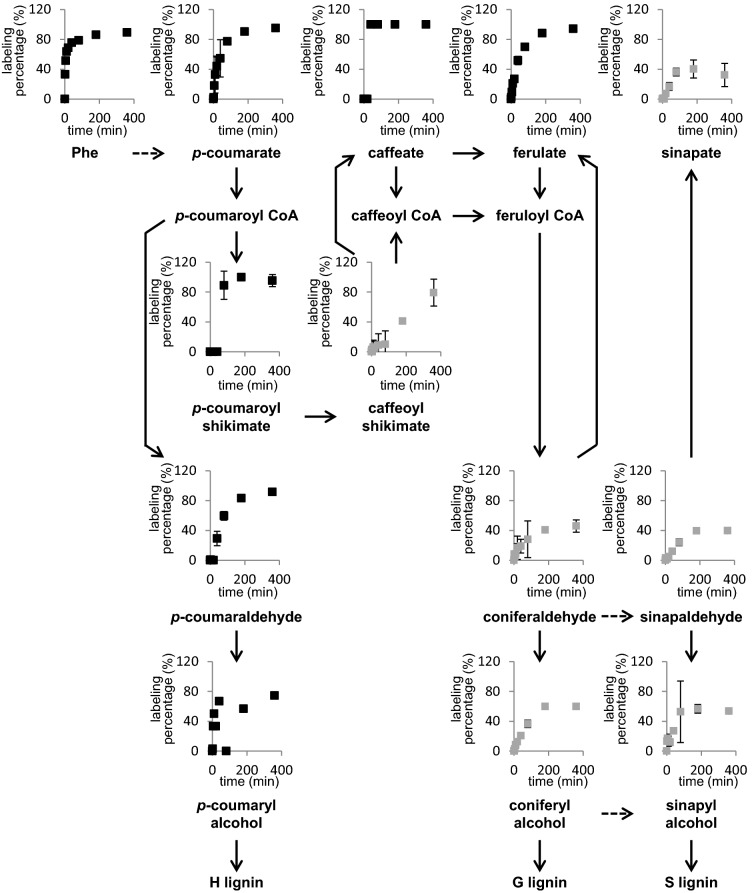
Labeling percentage of measured soluble phenylpropanoids from the base of Arabidopsis stems supplied with 1 mM [^13^C_6_]-Phe over the feeding time course. The plot of each metabolite was placed above its name on the pathway. Dashed lines mean multiple steps. Data represent mean ± SD (n = 3). Black squares indicate these metabolites were in the first group of the hierarchical clustering in Figure S4, and grey squares indicate these metabolites were in the second group

Using the established system, we fed 4-week-old wild-type Arabidopsis stems with 1.0 mM [^13^C_6_]-Phe and sampled basal 0–2 cm fragments over a time course of 360 min. [^13^C_6_]-Phe was used at a concentration of 1 mM to achieve high accumulation of labeled downstream phenylpropanoids for accurate quantification. Phe and the soluble phenylpropanoid intermediates were quantified by LC/MS–MS [33]. Supplied Phe was rapidly taken up by the stems as 33% of the entire Phe pool was labeled after 2 min (Fig. 5). At this time point downstream products including *p*-coumarate and ferulate were also labeled. After 40 min, caffeate, *p*-coumaraldehyde, coniferaldehyde, sinapaldehyde, and the corresponding monolignols were all labeled indicating that exogenous [^13^C_6_]-Phe was rapidly transported into lignifying cells for phenylpropanoid metabolism.

During the labeling period of 360 min, Phe and intermediates in the early part of the pathway rapidly approached isotopic steady state, whereas downstream metabolites showed a slower increase in labeling enrichment (Fig. 5). To achieve a quantitative overview of the labeling kinetics, we used hierarchical clustering to analyze the isotope enrichment profiles of the compounds measured. The hierarchical clustering based on squared Euclidian distance grouped the labeling percentage of phenylpropanoids into two patterns (Additional file 1: Figure S4). The first group contained Phe, *p*-coumarate, caffeate, ferulate, *p*-coumaraldehyde, *p*-coumaryl alcohol, and *p*-coumaroyl shikimate each of which exhibited rapid isotope enrichment in the first 80 min and a high final labeling of approximately 90%, except for *p*-coumaryl alcohol, which was 74%. In contrast, the second group including caffeoyl shikimate, coniferaldehyde, sinapaldehyde, coniferyl alcohol, sinapyl alcohol, and sinapate all showed slower increases in labeling, reaching less than 60% final labeling except for caffeoyl shikimate, which was slightly higher. These results are consistent with precursor-product relationships [38], where compounds closer to the fed substrate [^13^C_6_]-Phe are labeled faster and to a higher percentage than downstream product or than products with large endogenous pools. Furthermore, the efficient labeling of *p*-coumaryl alcohol provides an alternative or additional explanation for the high labeling of H-lignin that we previously observed (Fig. 2).

**Fig. 6:**
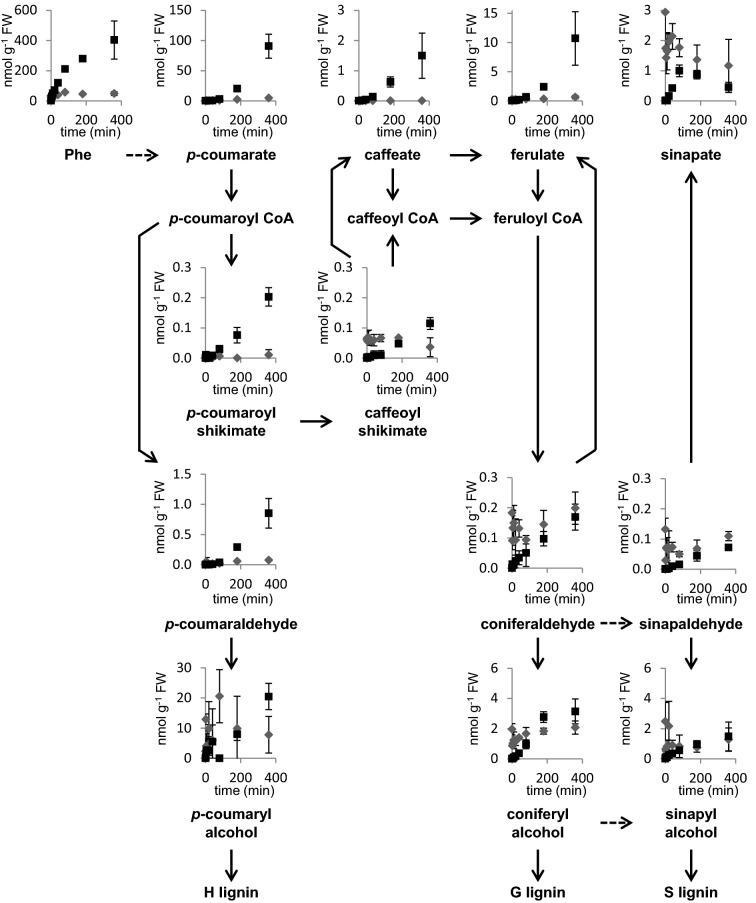
Metabolic profiles of soluble phenylpropanoids from the base of Arabidopsis stems supplied with 1 mM [^13^C_6_]-Phe over the feeding time course. Endogenous (grey) and ^13^C_6_ labeled (black) compounds were quantified with LC/MS–MS and normalized to fresh weight of stem tissue. The sum of endogenous and labeled concentrations of each metabolite can be found in Figure S6. The plot of each metabolite measured was placed above its name on the pathway. Dashed lines mean multiple steps. Data represent mean ± SD (n = 3)

In contrast to the pattern of isotopic enrichment observed for early pathway intermediates, the accumulation of labeled Phe and, except for sinapate, all downstream metabolites constantly increased and did not reach a metabolic steady state over the feeding period. Endogenous (unlabeled) Phe increased from 33 to around 50 nmol g FW^−1^ after 80 min, while [^13^C_6_]-Phe rapidly accumulated and reached approximately 400 nmol g FW^−1^ after 360 min, resulting in a labeling percentage of 89% (Fig. 6). Similarly, endogenous *p*-coumarate and ferulate concentrations significantly increased over the time course. [^13^C_6_]-*p*-coumarate and [^13^C_6_]-ferulate accumulated to 90 and 10.7 nmol g FW^−1^ after 360 min, approximately 20 times their endogenous levels (Fig. 6). Neither endogenous cinnamate nor [^13^C_6_]-cinnamate were detected in the stem tissue, even when high concentrations of [^13^C_6_]-Phe and [^13^C_6_]-*p*-coumarate were observed, possibly because cinnamate 4-hydroxylase (C4H) efficiently catalyzes cinnamate hydroxylation such that the level of its substrate remains below the limits of detection. Endogenous caffeate was at or near the detection limits in our experiment, however [^13^C_6_]-caffeate could be quantified readily after 40 min of feeding and reached 1.5 nmol g FW^−1^ after 360 min (Fig. 6). Unlike the other hydroxycinnamic acids, endogenous sinapate concentration was not changed during the feeding process, and [^13^C_6_]-sinapate reached steady state after 80 min, at a level lower than the unlabeled sinapate (Fig. 6). The pool size of endogenous unlabeled *p*-coumaraldehyde showed about a threefold increase after 360 min, while unlabeled *p*-coumaryl alcohol’s concentration did not change. Similarly, unlabeled coniferyl alcohol, sinapyl alcohol and their corresponding aldehydes did not change in abundance. The accumulation of these labeled aldehydes and alcohols were relatively modest (Fig. 6).

**Fig. 7:**
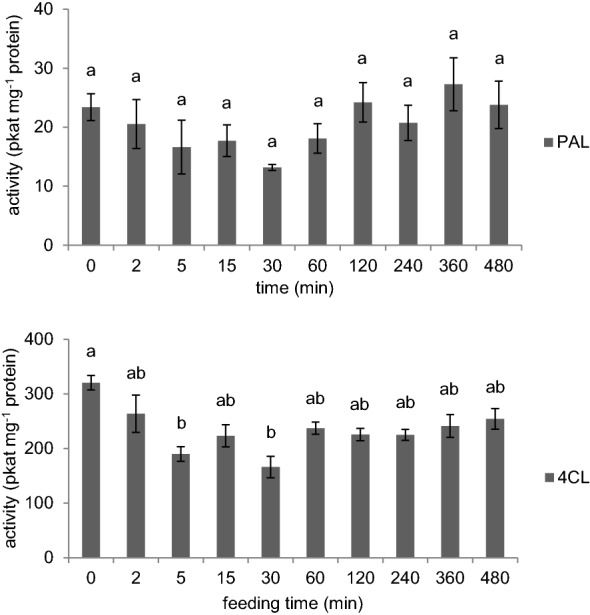
PAL and 4CL activities in the base of wild-type stems over the feeding course. Activities of PAL and 4CL were measured from the basal 0–2 cm of 4-week-old Arabidopsis stems fed with 1 mM Phe. Data represent mean ± SD (n = 3). One-way ANOVA was tested for PAL and 4CL assays respectively and statistical difference by Tukey HSD test (*p* < 0.01) was indicated by different letters

To test if the activities of biosynthetic enzymes are affected by the elevated phenylpropanoid levels, we determined the activities of PAL and 4CL in the stem tissue fed with 1 mM Phe over the time course. As shown in Fig. 7, PAL and 4CL activities both stayed constant even when the feeding period was extended to 480 min. This result suggests that increased levels of phenylpropanoids did not induce PAL or 4CL activities during the feeding process.

### Isotopic label is incorporated into lignin in stems fed with [^13^C_6_]-Phe

**Fig. 8:**
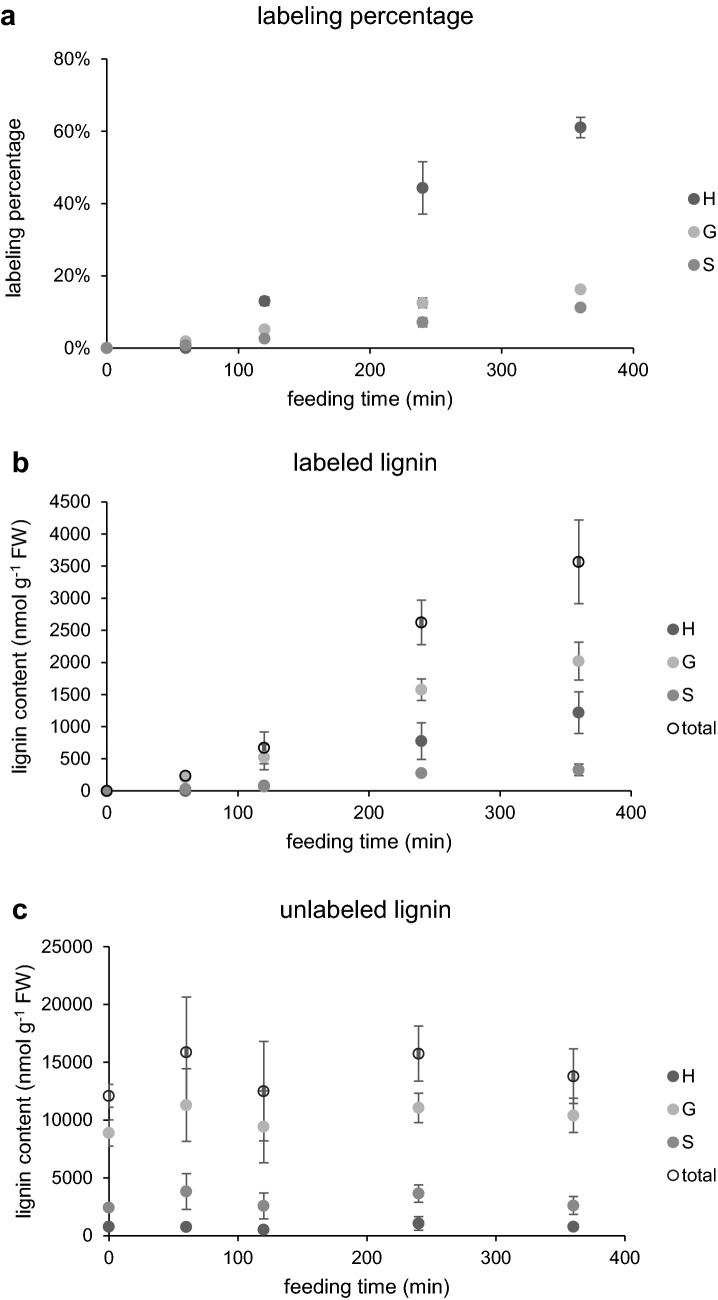
Analysis of lignin monomers in excised Arabidopsis stems fed with [^13^C_6_]-Phe. Lignin was analyzed by DFRC/GC/FID/MS from the base of 4-week-old wild-type Arabidopsis stems fed with 1 mM [^13^C_6_]-Phe for 0, 60, 120, 240, or 360 min. Labeling percentage (**a**) of each monomer was calculated using the labeled monomer (**b**) divided by the sum of labeled and unlabeled (**c**). Data represent mean ± standard deviation (n = 3)

To examine the isotopic labeling of lignin in the feeding experiment, we measured lignin composition by DFRC/GC using flame ionization detection (FID), and the labeling percentage of each released monomer by DFRC/GC/MS in stems fed with 1 mM [^13^C_6_]-Phe. G and S lignin released monomers were labeled in the primary stems after 60 min, while labeled H lignin was observed after 120 min (Fig. 8a). In samples where labeled H lignin was detected, its labeling percentage was more than twofold that of G or S lignin. This result is consistent with the observation that H lignin was labeled to a much higher level in whole stems fed for 48 h (Fig. 2). Next, we wanted to use the isotopic labeling of DFRC products to calculate the rate of lignin deposition during the feeding experiment. Because DFRC only detects lignin subunits that are exclusively β-O-4 linked [35], we measured lignin content by both the DFRC and the acetyl bromide methods and found that total lignin analyzed by the latter was 21.3-fold (n = 15) of DFRC lignin. We then used this value as a conversion factor to estimate lignin deposition using the DFRC data alone. A closer analysis of labeled H lignin content showed a rapid increase from near 80 nmol g FW^−1^ at 120 min to over 1200 nmol g FW^−1^ at 360 min (Fig. 8b). This large accumulation contributed to the high labeling percentage of 60% in addition to the small amount of endogenous unlabeled H lignin (Fig. 8a). Labeled G, and S lignin was deposited in a linear fashion over the time course at rates of approximately 6 and 1 nmol g FW^−1^ min^−1^, respectively. It is interesting that the ratio of deposition rates of labeled G and S lignin (6.1:1) differed from the G/S ratio of unlabeled lignin (3.6:1) measured at 0 min (Fig. 8c). The disproportional synthesis of labeled lignin subunits suggested that flux distribution towards different branches was affected by increased supply of the common precursor Phe. The total labeled lignin constituted by all three monomers showed a synthesis rate of 9.8 nmol g FW^−1^ min^−1^ in the sampled stem fragments. Because the changes in the sum of pre-existing lignins and the newly synthesized lignins deposited during the 360 min feeding experiment were within the standard deviation range of the DFRC results, the analysis of unlabeled lignin content showed no changes over the time course (Fig. 8c).

## Discussion

We developed a stable isotope feeding system to measure phenylpropanoid flux in intact Arabidopsis stems. In comparison to previous tracer experiments for lignin biosynthesis using cell suspension cultures [39, 40], the whole stems in our system continue their normal growth and development (Fig. 1), and conduct in situ phenylpropanoid metabolism and lignification (Fig. 2). The excised wild-type stems, resembling the plants growing in soil, consume supplied [^13^C_6_]-Phe that rapidly and efficiently labels the soluble phenylpropanoid intermediates and lignin over a time course of 360 min. The measurement of metabolites together with enzyme activities for lignin biosynthesis will provide valuable *in planta* input for analysis of flux through the phenylpropanoid pathway.

Application of isotope labeling has a long history in investigation of phenylpropanoid metabolism. Half a century ago, ^14^C labeled precursors were fed to various plants and cell suspension cultures to solve the structure of the pathway [39, 40]. Recently, the widespread use of MS technology has made it feasible to employ stable isotope labeling for the systematic estimation of metabolic fluxes in biochemical networks [3]. To analyze the flux through the branched lignin biosynthetic pathway in growing plants, an optimal feeding system needs to maintain physiologically normal conditions and active lignification. To this end, we chose intact Arabidopsis stems, where lignin is primarily deposited, excised from plants grown in soil. Previously, xylem sap extracted from plants was used as liquid medium to feed isotope labeled compounds to shoots of various plants [30, 31]. Because xylem sap contains amino acids including Phe [31, 41], we utilized a defined MS medium that avoids interference with endogenous unlabeled Phe and is practical to prepare in large volumes. The medium is adequate for stems to grow (Fig. 1) and even to develop fertile seeds when fresh medium was provided to the stems every day (data not shown). As the cauline leaves were all left intact, their transpiration promoted the rapid absorption of liquid MS medium together with the [^13^C_6_]-Phe at a rate of 1.4 µL min^−1^ (Additional file 1: Figure S3). When fed with 1 mM [^13^C_6_]-Phe, each stem on average took up approximately 500 nmol labeled Phe after 360 min, equivalent to 10% of the carbon fixed by photosynthesis in an Arabidopsis rosette of 200 mg in the same amount of time [5]. At the end of the feeding experiment, the stem base (approximately 30 mg) deposited over 3.5 µmol g FW^−1^ labeled lignin (Fig. 8), accounting for more than 20% of the labeled Phe absorbed. This measurement of the end products gives an estimation of total flux entering the pathway.

The Arabidopsis stem is a complex organ that comprises different tissues and cells, each of which differs in the level of phenylpropanoids they synthesize and the nature of the end products they accumulate. To find a suitable tissue for analysis, we assessed phenylpropanoid metabolite content and enzyme activities along the inflorescence stems and throughout the feeding process. In these experiments we found that the base of the stems contained the highest levels of labeled precursor and activities of PAL and 4CL (Fig. 3), demonstrating a high phenylpropanoid flux, consistent with the lignin deposition pattern observed in Arabidopsis [42].

The feeding of [^13^C_6_]-Phe to Arabidopsis stems showed rapid incorporation of label into downstream phenylpropanoid metabolism. Like previous studies done in various plant systems [43–46], we observed quick and high accumulation of labeled hydroxycinnamic acids (Fig. 6). The labeling kinetics of *p*-coumarate and ferulate followed that of Phe, indicative of rapid isotope incorporation. Interestingly, the labeling percentage profiles of all phenylpropanoids measured showed two different patterns apparently based on whether or not their synthesis was dependent on the enzyme *p*-coumaroylshikimate 3′-hydroxylase (C3′H). The compounds that do not require C3′H for their synthesis displayed rapid increases in labeling enrichment and reach isotopic steady state within 80 min of feeding. In contrast, C3′H-dependent compounds were mostly labeled more slowly (Fig. 5). One possible explanation is that the relatively large pools of endogenous unlabeled coniferyl alcohol, sinapyl alcohol, and sinapate in the stems render the labeling slow, but it cannot explain the results seen with coniferaldehyde and sinapaldehyde which had much less accumulation (Fig. 6). Another possibility is that a slow (often rate-limiting) enzymatic step provokes high labeling of upstream intermediates and causes slow and low labeling in downstream products [4, 47]. C3′H is a cytochrome P450-dependent monooxygenase, a class of enzymes generally known to have low turnover [48, 49]. In the feeding experiments, the substantial accumulation of *p*-coumaroylshikimate indicates that C3′H might be saturated, becoming a limiting step for lignification. Caffeate and ferulate are two exceptions in the first group as both are 3′-hydroxylated but exhibited a labeling pattern like Phe and *p*-coumarate. The labeling percentage of ferulate is higher than its shikimate ester precursors, which seems to suggest *p*-coumarate is a close upstream precursor for caffeate and ferulate and that its conversion to these two downstream intermediates bypasses the HCT/C3′H/HCT/CSE steps. Supporting this model, it was shown that C4H and C3′H from poplar form a protein complex and that the recombinant proteins can catalyze the hydroxylation of *p*-coumarate in vitro [50].

Lignification in plants is tightly regulated transcriptionally and post-translationally to control carbon allocation into this major metabolic sink. Recent studies have shown that the expression of *PAL* genes is regulated by Mediator and transcriptional factors [51, 52] and that Kelch repeat F-box proteins mediate the degradation of PAL enzymes via the proteasome complex [53]. PAL has often been considered as the rate-limiting step in lignin biosynthesis [54] and this may well be the case when PAL is down-regulated. In contrast, our study shows that in wild-type Arabidopsis plants, PAL is present in excess and the availability of Phe instead determines lignin deposition, a factor that has been undervalued in the past. Before feeding, stems had developed for 6 days in soil and deposited approximately 12 µmol g FW^−1^ of lignin. When fed with 1.0 mM [^13^C_6_]-Phe for only 360 min, stems synthesized over 3 µmol g FW^−1^ additional, labeled, lignin, accounting for 25% of the previous total, showing a higher lignification rate than would occur under normal, unfed, conditions. Given that PAL activity remains unaltered during the feeding period (Fig. 7), these data indicate that an enhanced supply of Phe increases lignin biosynthesis in plants, and that PAL is not saturated with endogenous Phe in vivo under normal conditions. Consistent with these observations, it has been reported that the Arabidopsis *pal1* mutant has a 40% reduction in PAL activity but deposits wild-type levels of lignin [55, 56], supporting the idea that PAL in wild-type plants does not perform at maximal velocity and is not the rate-limiting enzyme.

Many studies have reported that lignin monomer composition is altered in transgenic plants or mutants when flux into or within the phenylpropanoid pathway is reduced. In addition to decreased lignin content, an increased S/G ratio was seen in mutants with reduced levels of PAL, C4H, 4CL, or arogenate dehydratase, an enzyme in Phe biosynthesis [55, 57–59]. It has been suggested that these changes occur when enzymes with markedly different kinetic characteristics that act at pathway branch points compete for a common intermediate, thus leading to altered flux partitioning when the two enzymes’ substrate concentration is decreased [58]. In contrast, experimental evidence to show the opposite change, increased total influx leading to a decreased S/G ratio, has not previously been reported. As this model predicts, we found that enhanced supply of Phe resulted in increased H lignin deposition and comparatively reduced S lignin synthesis (Fig. 8). In plants, cinnamoyl CoA reductase (CCR) competes with hydroxycinnamoyl CoA: shikimate hydroxycinnamoyl transferase (HCT) for *p*-coumaroyl CoA to drive flux towards H lignin (Additional file 1: Figure S1). It has been suggested that HCT associates with the complex of C4H and C3′H on the endoplasmic reticulum membrane, forming a metabolon that efficiently directs carbon towards G and S lignin in plants under normal conditions [60]. This is suggested to occur even though the reported *K*_M_ of Arabidopsis CCR1 (2.27 µM towards *p*-coumaroyl CoA, [61]) is smaller than the *K*_M_ of HCT from tobacco (600 µM, 23]) and the maximal activity of CCR (200 pkat mg^−1^ protein) in Arabidopsis stem extracts is higher than that of HCT (35 pkat mg^−1^ protein) using *p*-coumaroyl CoA as substrate (unpublished data). When *p*-coumaroyl CoA accumulates and C3′H in the metabolon becomes saturated, CCR likely outcompetes HCT and generates higher levels of *p*-coumaraldehyde, which ultimately results in more H lignin. Similarly, ferulate 5-hydroxylase, the last cytochrome P450-dependent monooxygenase in the pathway, typically competes effectively with cinnamyl alcohol dehydrogenase and the yet-to-be identified transporter of coniferyl alcohol to synthesize S lignin (Additional file 1: Figure S1) [62]. When high concentrations of coniferaldehyde and coniferyl alcohol are present, proportionally more flux escapes 5-hydroxylation leading to a decreased S/G ratio (Fig. 8). The precise control of flux by the enzymes at each branch points will be better understood by a more sophisticated and mechanistic mathematic model that includes the kinetics of the enzymes involved.

## Conclusions

In summary, we established an experimental feeding system to supply intact Arabidopsis stems with [^13^C_6_]-Phe to investigate the metabolic flux towards lignin. Our analysis revealed that the availability of Phe determines lignin deposition rate and can alter distribution of flux towards three monolignols. The soluble phenylpropanoid metabolite and lignin measurements from dynamic isotope labeling experiments can be input for mathematical modeling of metabolic fluxes to quantitatively unravel the control of flux and to explore its regulation. In addition to the measurement of lignin biosynthetic flux in wild-type Arabidopsis, we envision a wider application of this stem feeding system. For example, mutants with defects in lignification can be fed with labeled precursor in parallel with the wild type to explore how genetic perturbation affects flux distribution within the phenylpropanoid pathway. Furthermore, given the abundance of Arabidopsis mutants in myriad biochemical pathways, isotope labeled substrates could be administered with this system such that their flux distribution could be described but in this case, reoptimization of the selected tissue would be warranted.

## Methods

### Plant material and growth conditions

*Arabidopsis thaliana* (Columbia-0) were grown in soil at 23 °C under light intensity of 150 µE m^−2^ s^−1^ in a photoperiod of 16-h light and 8-h dark in a growth chamber. Primary stems of 4-week-old plants were used for feeding experiments and enzyme activity assays.

### Feeding

Arabidopsis stems were fed with liquid MS medium containing [^13^C_6_]-Phe in 1.5 mL tubes in the same growth chamber as they were grown into maintain the same environmental conditions. [^13^C_6_]-Phe was filter-sterilized before being added into autoclaved MS medium. A steel nail was heated and used to form a hole in the lid of tubes so that the stems could be inserted through it. Plants were next removed from soil, and then the inflorescence stem was cut immediately above the rosette with a double-edged razor blade under water. The excised stem was immediately transferred into incubation solution and placed on a rack in the growth chamber (Additional file 1: Figure S2). Tubes were preloaded with 0.5 mL medium and refilled with fresh medium when necessary. After the specified incubation period, stem fragments were rinsed three times with water to remove [^13^C_6_]-Phe on the surface and frozen in liquid nitrogen. Ten stem fragments were harvested as one sample, and three biological replicates were generated for each time point. Depending on the biological questions asked and quantification methods employed, more biological replicates or stem tissue *per* sample may be optimized to achieve accurate measurements of metabolites. Samples were stored at − 70 °C until extraction.

### Stem growth measurement

Stems of 4-week-old plants were cut under water as described above and used for growth analysis. Stems were photographed alongside a ruler immediately and after 3, 6, 9, 12, 24, 27, 30, 33, 36, and 48 h. Measurements of stem length from the base to the apical meristem were done using ImageJ in comparison to the ruler included in the photograph.

### LC/MS–MS analysis of soluble metabolites

Soluble metabolites were analyzed with LC/MS–MS following the method developed by Jaini et al. [33]. Briefly, stem tissue was extracted in 75% methanol (v/v) (at 10 µL mg FW^−1^) for 2 h at 65 °C, and supernatant was collected after centrifugation for 20 min at 16,000×*g*. To concentrate the samples, 500 µL of supernatant was dried in speed-vac and then re-dissolved in 50 µL 50% methanol (v/v). Samples were analyzed by LC/MS–MS using a Zorbax Eclipse C8 column (150, 4.6 mm, 5 µm, Agilent Technologies, Santa Clara, CA, USA) and ammonium acetate (pH 5.6) and acetonitrile/H_2_O/HCOOH (9.8/2/0.2) as mobile phase. Metabolite detection was achieved with a QTrap 5500 triple quadruple mass spectrometer (AB Sciex, Redwood City, CA,USA), equipped with an ESI-Turbolon-spray to operate in negative ion mode. Multiple reaction monitoring mode was used to quantity compounds. Standards of Phe, cinnamate, *p*-coumarate, caffeate, ferulate, sinapate, shikimate, *p*-coumaraldehyde, coniferaldehyde, sinapaldehyde, *p*-coumaryl alcohol, coniferyl alcohol, sinapyl alcohol, *p*-coumaroyl shikimate, caffeoyl shikimate were analyzed by LC/MS–MS to generate calibration curve to quantify the soluble metabolites. The same curve was used to quantify both unlabeled and labeled isotopologues. The labeling percentage was calculated with the following equation:

$$Labeling\, percentage = \frac{{\left[ {Labeled\, compound} \right]}}{{\left[ {Labeled\, compound} \right] + \left[ {Unlabeled \,compound} \right]}}*100\%$$

The hierarchical clustering analysis of isotope enrichment profiles of all measured metabolites was performed using averaged labeling percentage data of all time points. Squared Euclidian distance was computed and clustered in R [63].

### DFRC/GC/FID and DFRC/GC/MS

Stems were analyzed for lignin content following the method established by Lu and Ralph 1997 [35]. Briefly samples were ground in liquid nitrogen washed five times with 10 mL 70% ethanol (v/v) at 80 °C and once with acetone. The dried cell wall residue was weighed and dissolved in 2.5 mL of acetyl bromide/glacial acetic acid (20:80 v/v) mixture containing 0.2 mg internal standard (4 4′-ethyldenebisphenol) overnight at room temperature. The mixture was dried under nitrogen gas then dissolved in 2 mL of dioxane/glacial acetic acid/H_2_O (50:40:10 v/v/v). 50 mg zinc dust was added to the mixture vortexed stirred for 25 min and applied to a solid phase extraction column (Discovery^®^ DSC-18 SPE tube) pre-conditioned with 95% ethanol (v/v) and H_2_O. The column was washed with 5 mL 25% ethanol (v/v) and DFRC reactions products were eluted with 2.5 mL 96% ethanol (v/v) then dried under nitrogen gas. The sample was acetylated with 0.5 mL acetic anhydride/pyridine (60/40 v/v) overnight and dried again under nitrogen gas. The sample was then dissolved in 200 µL dichloromethane and 1 µL of the final product analyzed by GC/FID or GC/MS.

### Acetyl bromide lignin analysis

Total lignin content in the stem tissue was quantified using acetyl bromide lignin analysis method described in 64] with minor revisions. Briefly the basal 0–2 cm fragments of stems of 4-week-old Arabidopsis were harvested and 15 biological replicates were included for analysis. The stem tissue was ground in liquid nitrogen and washed in 0.1 M sodium phosphate buffer (pH 7.2) at 50 °C for 30 min followed by five washes with 10 mL 70% ethanol (v/v) at 80 °C and once with 2 mL acetone. The dried cell wall residue was weighed and dissolved in 2.5 mL of acetyl bromide/glacial acetic acid (25:75 v/v) overnight at room temperature. A control without cell wall residue was included. The dissolved samples were completely transferred into 50 mL volumetric flasks containing 2.5 mL 2 M NaOH and 12 mL acetic acid. 0.5 mL freshly prepared 7.5 M hydroxylamine hydrochloride was added into each sample followed by 35 mL acetic acid. The sample was mixed and allowed to settle before the volume was brought to 50 mL with acetic acid. Absorbance at 280 nm was measured on spectrophotometer using the control as blank. Extinction coefficient 23.20 g^−1^ L cm^−1^ was used to calculate total lignin content.

### PAL and 4CL enzyme assays

Stem tissue was harvested and frozen in liquid nitrogen. Crude protein was extracted from ground tissue with Tris–HCl buffer at pH of 7.8 and desalted on a gel filtration column (Sephadex™ G-50 fine GE Healthcare). PAL and 4CL assays were conducted following the method in Klempien 2010 65]. Each PAL assay contained 100 mM Tris–HCl buffer pH 7.5 5 mM Phe and 5 µL protein extract in a final volume of 50 µL. The reactions were incubated at 23 °C for 120 min. The 4CL assay contained 100 mM Tris–HCl buffer pH 7.5 5 mM MgCl_2_ 5 mM ATP 1 mM *p*-coumarate 0.3 mM CoA and 2 µL protein extract in a final volume of 40 µL. Each reaction was incubated at 23 °C for 20 min. Assay products were quantified on HPLC with cinnamate and synthesized *p*-coumaroyl CoA as standards respectively. Protein concentrations were measured with Bradford assay using bovine serum albumin as standard.

## Additional file

**Additional file 1.** **Figure S1.** A simplified pathway illustrating the enzymes and metabolites involved in lignin biosynthesis. PAL, phenylalanine ammonia lyase; C4H, cinnamate 4-hydroxylase; 4CL, 4-coumarate CoA ligase; HCT, hydroxycinnamoyl CoA:shikimatehydroxycinnamoyl transferase; C3′H, p-coumaroyl shikimate 3′-hydroxylase; CSE, caffeoyl shikimate esterase; CCoAOMT, caffeoyl CoA O-methyltransferase; F5H, ferulate5-hydroxylase; COMT, caffeic acid O-methyltransferase; CCR, cinnamoyl CoA reductase; CAD, cinnamyl alcohol dehydrogenase. SALDH, sinapaldehydedehydrogenase. **Figure S2.** Excised stems incubated in tubes with MS medium in growth chamber. (A) An Arabidopsis stem was excised and placed into a 1.5 mL tube containing liquid MS medium. (B) Arabidopsis stems incubated in MS medium were placed in a rack to perform feeding experiment (picture taken from side). (C) Stems were sitting away from each other to mimic their growth in the soil (picture taken from top). **Figure S3.** Medium absorbed by the excised stems during the feeding process. The loss of medium from each tube with an excised stem was measured after feeding for 0, 40, 90, 180, and 240 min. Data represented mean ± SD (n = 45). **Figure S4.** Hierarchical clustering of labeling percentage profiles of soluble phenylpropanoids from the base of Arabidopsis stems supplied with 1 mM [^13^C_6_]-Phe over the feeding time course. The averaged labeling percentage data of each metabolite over the time course from Figure 5 were clustered based on squared Euclidian distance. **Figure S5.** Metabolic profiles of soluble phenylpropanoids from the base of Arabidopsis stems supplied with 1 mM [^13^C_6_]-Phe over the feeding time course. Sum of endogenous and ^13^C_6_ labeled compounds was quantified with LC/MS-MS and normalized to fresh weight of stem tissue. The plot of each metabolite measured was placed above its name on the pathway. Dashed lines mean multiple steps. Data represent mean ± SD (n = 3).
