# Supplementary material for: A 13C isotope labeling method for the measurement of lignin metabolic flux in Arabidopsis stems
Source: Plant Methods. 2018 Jun 23;14:51. doi: 10.1186/s13007-018-0318-3 (PMC6015466; doi:10.1186/s13007-018-0318-3)
Supplement: Supplementary file 1 — Additional file 1. Figure S1. A simplified pathway illustrating the enzymes and metabolites involved in lignin biosynthesis. PAL, phenylalanine ammonia lyase; C4H, cinnamate 4-hydroxylase; 4CL, 4-coumarate CoA ligase; HCT, hydroxycinnamoyl CoA:shikimatehydroxycinnamoyl transferase; C3′H, p-coumaroyl shikimate 3′-hydroxylase; CSE, caffeoyl shikimate esterase; CCoAOMT, caffeoyl CoA O-methyltransferase; F5H, ferulate5-hydroxylase; COMT, caffeic acid O-methyltransferase; CCR, cinnamoyl CoA reductase; CAD, cinnamyl alcohol dehydrogenase. SALDH, sinapaldehydedehydrogenase. Figure S2. Excised stems incubated in tubes with MS medium in growth chamber. (A) An Arabidopsis stem was excised and placed into a 1.5 mL tube containing liquid MS medium. (B) Arabidopsis stems incubated in MS medium were placed in a rack to perform feeding experiment (picture taken from side). (C) Stems were sitting away from each other to mimic their growth in the soil (picture taken from top). Figure S3. Medium absorbed by the excised stems during the feeding process. The loss of medium from each tube with an excised stem was measured after feeding for 0, 40, 90, 180, and 240 min. Data represented mean ± SD (n = 45). Figure S4. Hierarchical clustering of labeling percentage profiles of soluble phenylpropanoids from the base of Arabidopsis stems supplied with 1 mM [13C6]-Phe over the feeding time course. The averaged labeling percentage data of each metabolite over the time course from Figure 5 were clustered based on squared Euclidian distance. Figure S5. Metabolic profiles of soluble phenylpropanoids from the base of Arabidopsis stems supplied with 1 mM [13C6]-Phe over the feeding time course. Sum of endogenous and 13C6 labeled compounds was quantified with LC/MS-MS and normalized to fresh weight of stem tissue. The plot of each metabolite measured was placed above its name on the pathway. Dashed lines mean multiple steps. Data represent mean ± SD (n = 3). [file 13007_2018_318_MOESM1_ESM.pdf]

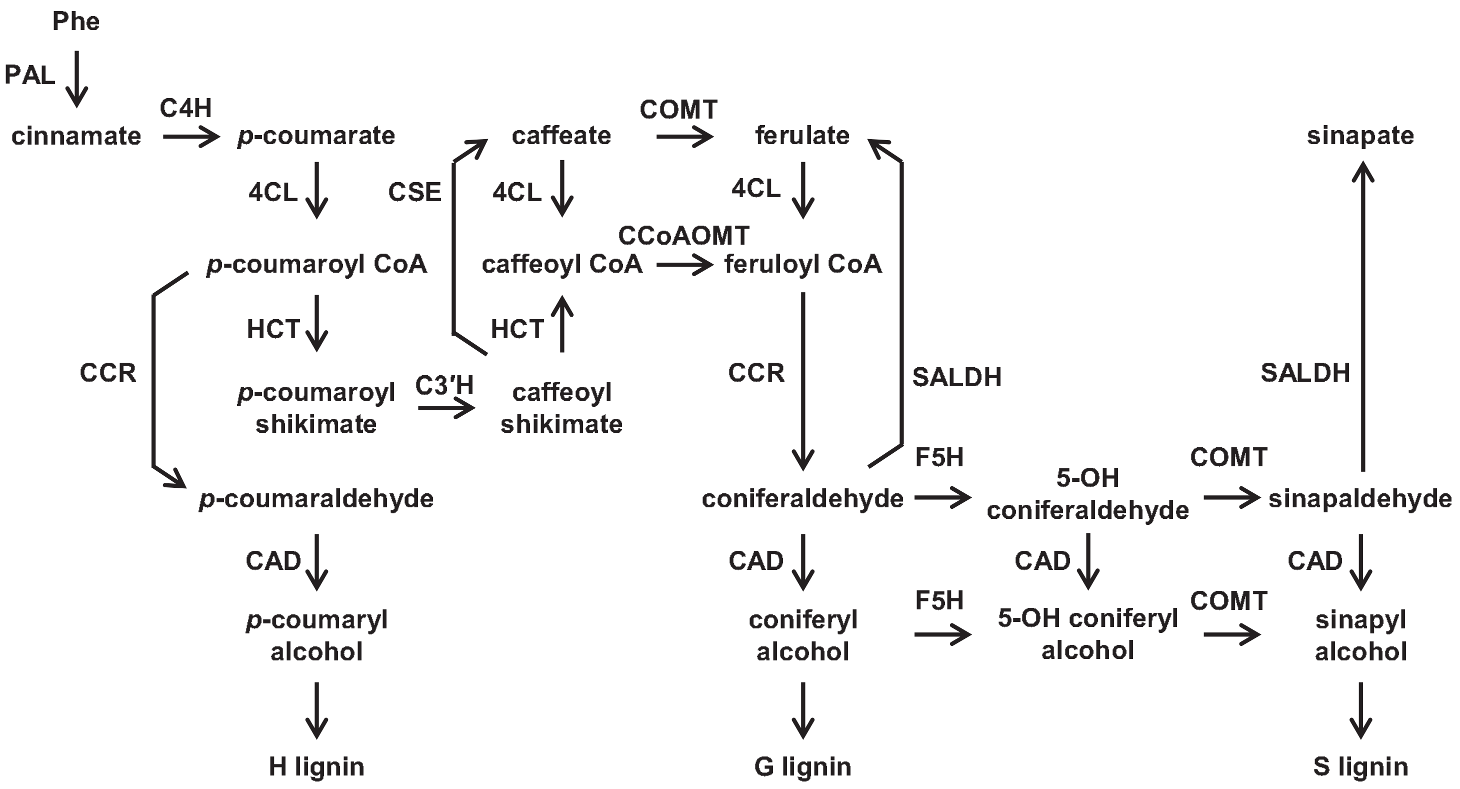

Figure S1. A simplified pathway illustrating the enzymes and metabolites involved in lignin biosynthesis. PAL, phenylalanine ammonia lyase; C4H, cinnamate 4-hydroxylase; 4CL, 4-coumarate CoA ligase; HCT, hydroxycinnamoyl CoA:shikimate hydroxycinnamoyl transferase; C3′H, *p*-coumaroyl shikimate 3′-hydroxylase; CSE, caffeoyl shikimate esterase; CCoAOMT, caffeoyl CoA *O*-methyltransferase; F5H, ferulate 5-hydroxylase; COMT, caffeic acid *O*-methyltransferase; CCR, cinnamoyl CoA reductase; CAD, cinnamyl alcohol dehydrogenase. SALDH, sinapaldehyde dehydrogenase.

A

B

C

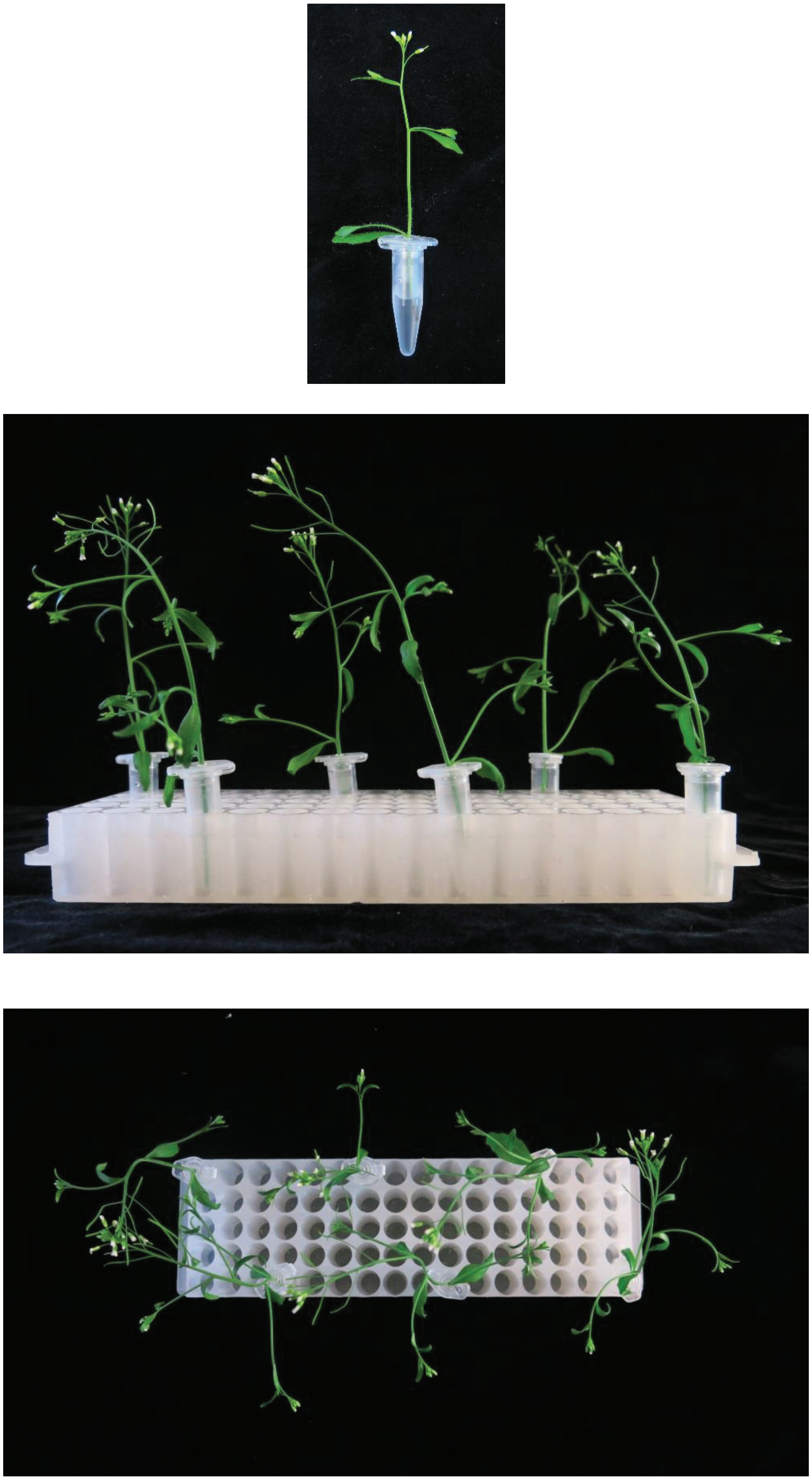

Figure S2. Excised stems incubated in tubes with MS medium in growth chamber.
(A) An Arabidopsis stem was excised and placed into a 1.5 mL tube containing liquid MS medium.
(B) Arabidopsis stems incubated in MS medium were placed in a rack to perform feeding experiment (picture taken from side).
(C) Stems were sitting away from each other to mimic their growth in the soil (picture taken from top).

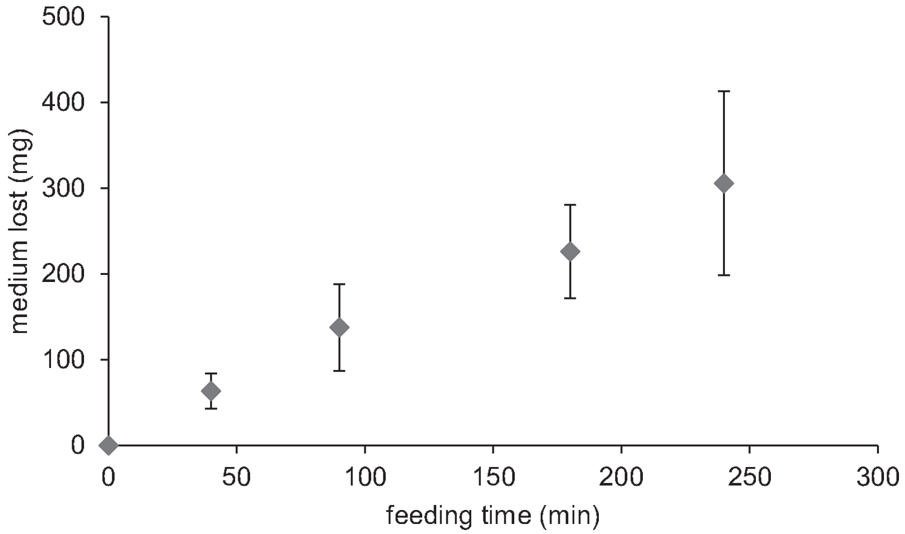

Figure S3. Medium absorbed by the excised stems during the feeding process. The loss of medium from each tube with an excised stem was measured after feeding for 0 min, 40 min, 90 min, 180 min, and 240 min. Data represented mean ± standard deviation (n = 45).

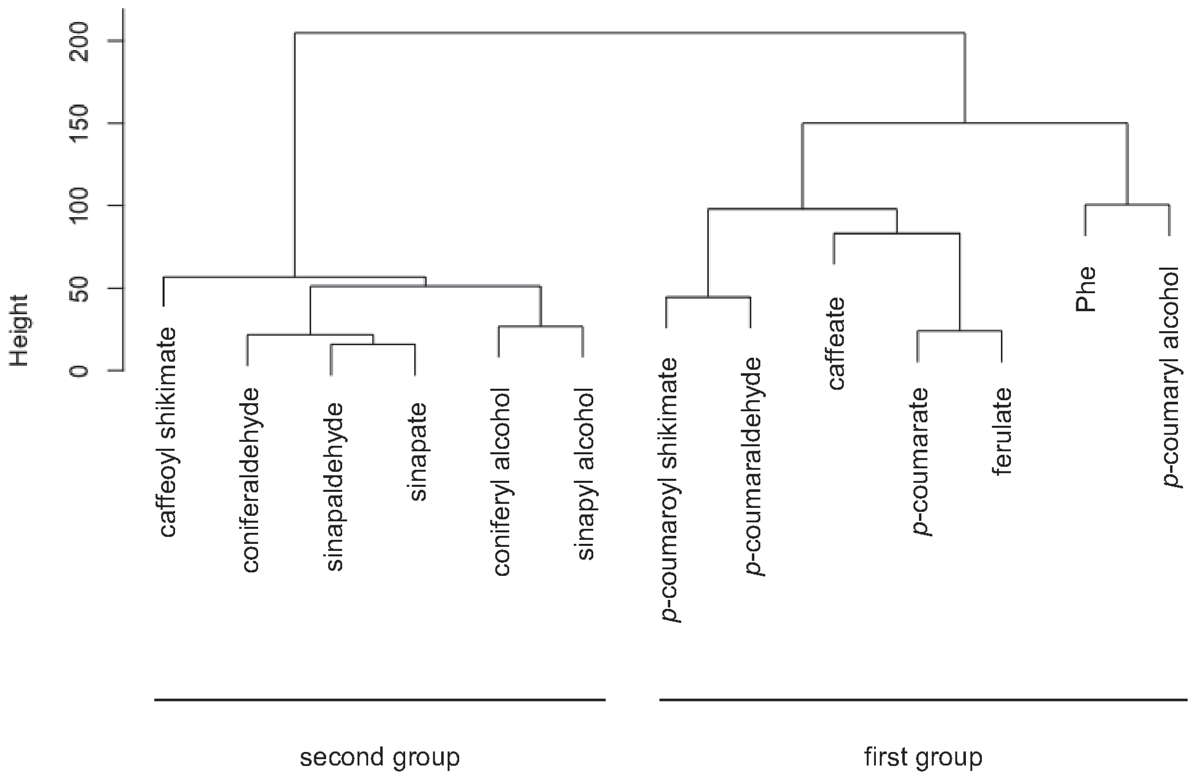

Figure S4. Hierarchical clustering of labeling percentage profiles of soluble phenylpropanoids from the base of Arabidopsis stems supplied with 1 mM [$^{13}C_6$]-Phe over the feeding time course. The averaged labeling percentage data of each metabolite over the time course from Figure 5 were clustered based on squared Euclidian distance.

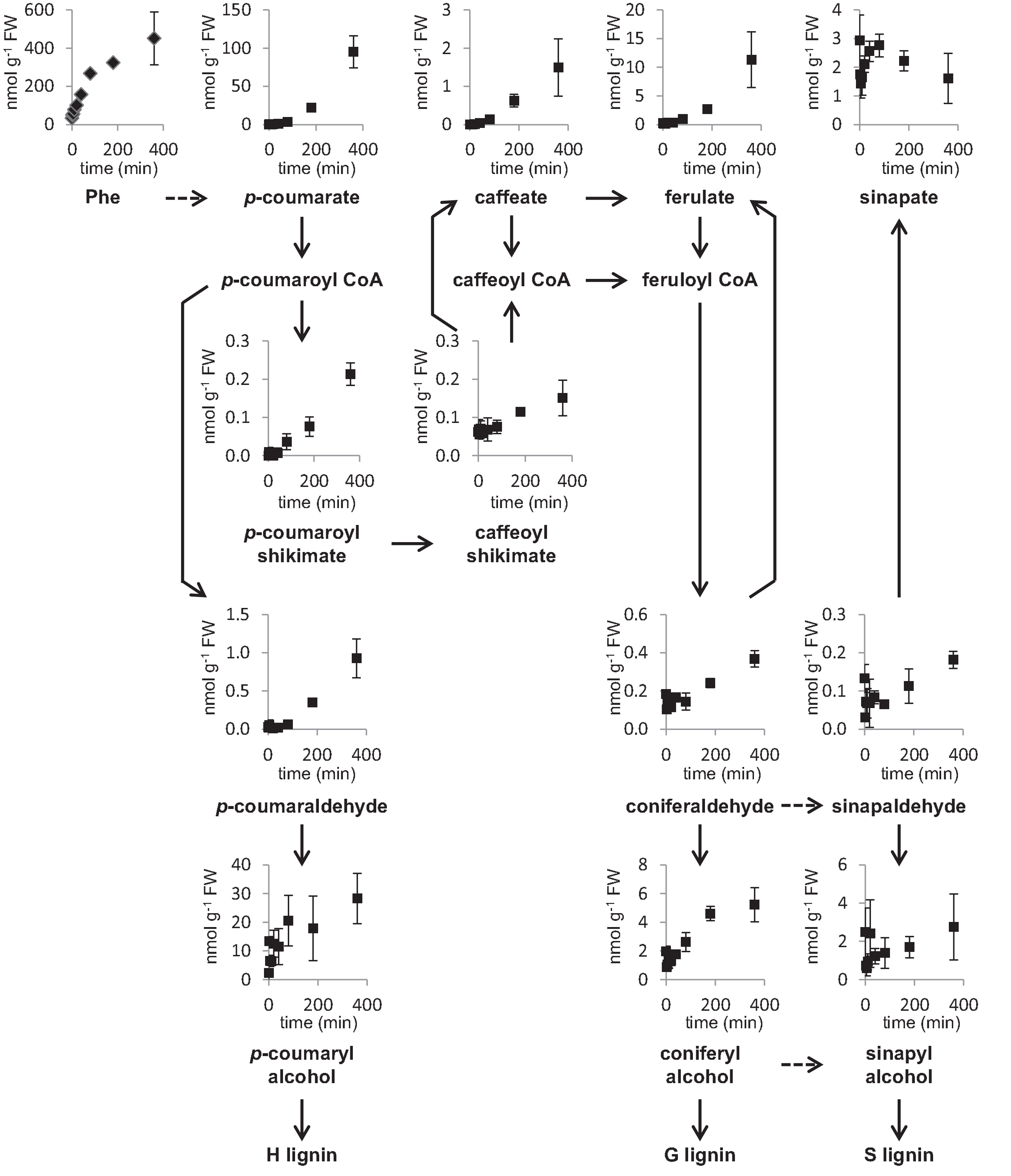

Figure S5. Metabolic profiles of soluble phenylpropanoids from the base of Arabidopsis stems supplied with 1 mM [$^{13}C_6$]-Phe over the feeding time course. Sum of endogenous and $^{13}C_6$ labeled compounds was quantified with LC/MS-MS and normalized to fresh weight of stem tissue. The plot of each metabolite measured was placed above its name on the pathway. Dashed lines mean multiple steps. Data represent mean ± SD (n=3).
